# Importance of time in therapeutic range on bleeding risk prediction using clinical risk scores in patients with atrial fibrillation

**DOI:** 10.1038/s41598-017-11683-2

**Published:** 2017-09-21

**Authors:** José Miguel Rivera-Caravaca, Vanessa Roldán, María Asunción Esteve-Pastor, Mariano Valdés, Vicente Vicente, Gregory Y. H. Lip, Francisco Marín

**Affiliations:** 1 Department of Cardiology, Hospital Clínico Universitario Virgen de la Arrixaca, Instituto Murciano de Investigación Biosanitaria (IMIB-Arrixaca), Murcia, Spain; 2 Department of Hematology and Clinical Oncology, Hospital Universitario Morales Meseguer, University of Murcia, Instituto Murciano de Investigación Biosanitaria (IMIB-Arrixaca), Murcia, Spain; 3 Institute of Cardiovascular Sciences, University of Birmingham, United Kingdom, and Aalborg Thrombosis Research Unit, Department of Clinical Medicine, Aalborg University, Aalborg, Denmark

## Abstract

Bleeding risk with vitamin K antagonists (VKAs) is closely related to the quality of anticoagulation in atrial fibrillation (AF) patients, reflected by time in therapeutic range (TTR). Here we compared the discrimination performance of different bleeding risk scores and investigated if adding TTR would improve their predictive value and clinical usefulness. We included 1361 AF patients stables on VKA for at least 6 months. Bleeding risk was assessed by the HAS-BLED, ATRIA, ORBIT and HEMORR_2_HAGES scores. Major bleeding events were recorded after a median of 6.5 years follow-up. In this period 250 patients suffered major bleeds. Comparison of receiver operating characteristic (ROC) curves demonstrated that HAS-BLED had the best discrimination performance, but adding the ‘labile INR’ criteria (i.e. TTR <65%) to ATRIA, ORBIT and HEMORR_2_HAGES increased their ability of discrimination and predictive value, with significant improvements in reclassification and discriminatory performance. Decision curve analyses (DCA) showed improvements of the clinical usefulness and a net benefit of the modified risk scores. In summary, in AF patients taking VKAs, the HAS-BLED score had the best predictive ability. Adding ‘labile INR’ to ATRIA, ORBIT and HEMORR_2_HAGES improved their predictive value for major bleeding leading to improved clinical usefulness compared to the original scores.

## Introduction

Oral anticoagulation (OAC) is highly effective reducing thromboembolism and mortality in patients with atrial fibrillation (AF)^1,2^. Given that OAC confers a risk of bleeding, various clinical risk scores have been proposed to help risk stratification^3^, such as HAS-BLED, ATRIA, HEMORR_2_HAGES and more recently, ORBIT (Supplementary Table 1).

One of the advantages of HAS-BLED is its simplicity, yet useful predictive capability for bleeding in users of VKA and non-VKA anticoagulants, aspirin or no antithrombotic therapy^4,5^. Importantly, HAS-BLED draws attention to the potentially modifiable bleeding risk factors, such as uncontrolled hypertension (‘H’ in HAS-BLED), concomitant use of aspirin/non-steroidal anti-inflammatory drugs (NSAIDs) or alcohol excess (‘D’ in HAS-BLED)^6,7^. HAS-BLED also takes into consideration the quality of anticoagulation control amongst VKA users (i.e. the ‘labile INRs’ criterion, often defined by the time in therapeutic range [TTR] <65% or similar indices, e.g. proportion of INRs in range (PINRR))^8^. In VKA users, good quality anticoagulation control is a cornerstone, given that the efficacy and safety of VKA is intimately related to TTR^4,9^. Indeed, both major bleeding and mortality rates are significantly higher with low TTR^10,11^. Despite the introduction of the Non-VKA Oral Anticoagulants (NOACs), the VKAs remain very widely used world-wide, and to have a simple bleeding risk score valid in VKA and non-VKA anticoagulants, aspirin or no antithrombotic therapy allows clinical application in all parts of the AF patient management pathway.

Based on clinical trial cohorts, other risk scores have been proposed, to be valid in VKA or NOACs users by not considering ‘labile INR’ as a criterion. In clinical trials, however, patients are often carefully selected and followed up regularly, whereas AF patients in ‘real world’ clinical practice tend to be older, with associated comorbidities and polypharmacy.

In the present study, we have compared the four AF-validated bleeding risk schemas in a large ‘real world’ cohort of AF patients over a long period of follow-up. Second, we tested if the predictive values of ATRIA, ORBIT and HEMORR_2_HAGES scores, and their clinical usefulness could be improved adding a labile INR criterion (defined as TTR <65%).

## Methods

From May 1, 2007 to December 1, 2007 we recruited consecutive patients with paroxysmal, persistent or permanent AF who had steady OAC with VKA (INR 2.0–3.0) for at least 6 months, in our single anticoagulation center from a tertiary Hospital in Murcia (Southeastern Spain). At baseline, all patients were receiving anticoagulation therapy with acenocoumarol (the commonest VKA used in Spain) and consistently achieved an INR between 2.0 and 3.0 during the previous 6 months (hence, TTR 100% for this cohort – to ensure baseline homogeneity and avoiding the bias produced by a low TTR at entry or initially unstable INRs especially in an inception cohort). Patients with prosthetic heart valves or AF due to mitral valve stenosis, recent acute coronary syndrome (ACS), stroke (ischemic or embolic), or any hemodynamic instability that led hospital admission or surgical intervention in the preceding 6 months were excluded.

At baseline, a complete medical history was recorded and stroke risk (CHADS_2_) and bleeding risk (HEMORR_2_HAGES) were calculated. Other risk scores (CHA_2_DS_2_-VASc for stroke risk; HAS-BLED, ATRIA and ORBIT for bleeding risk) were calculated retrospectively using the clinical variables available in our (prospectively collected) dataset. The TTR at 6 months after entry was calculated using the linear interpolation method of Rosendaal^12^. Good anticoagulation control was defined as a TTR >65%, based on recommendations of the National Institute for Health and Care Excellence (NICE)^13^. Anemia was defined as hemoglobin <13 g/L in men and <12 g/L in women.

Follow-up was performed by personal interview at each visit to the anticoagulation clinic and through medical records. During this period we recorded all bleeding events, which were categorized as major bleeding (primary endpoint) if they met the following 2005 International Society on Thrombosis and Haemostasis (ISTH) criteria^14^: fatal bleeding, and/or symptomatic bleeding in a critical area or organ, such as intracranial, intraspinal, intraocular, retroperitoneal, intra-articular or pericardial, or intramuscular with compartment syndrome, and/or bleeding causing a fall in hemoglobin level of 20 g.L^−1^ (1.24 mmol.L^−1^) or more, or leading to transfusion of two or more units of whole blood or red cells. Bleeding events, as well as other clinical outcomes, were identified, confirmed and recorded by the investigators.

This observational registry was approved by the Ethical Committee from University Hospital Morales Meseguer and was performed in accordance with the ethical standards laid down in the 1964 Declaration of Helsinki and its later amendments. All patients gave informed consent to participation in the study.

### Statistical analysis

Categorical variables are presented as counts and percentages. Continuous variables were tested for normality by the Kolmogorov-Smirnov test and presented as mean ± standard deviation (SD) or median and interquartile range (IQR), as appropriate. The Chi-squared test was used to compare proportions. Cox regression models were performed to determine the association between higher values (or high risk and medium/high risk when we analyzed as categories) of the bleeding risk scores and the occurrence of a major bleeding.

Kaplan-Meier estimates and analysis by the long-rank test were carried out to assess differences in event-free survival distributions between subgroups of bleeding risk categories. Receiver operating characteristic (ROC) curves were applied to evaluate the predictive ability (expressed as c-indexes) of the four AF-validated bleeding risk scores. Comparisons of ROC curves were carried out by DeLong *et al*. method^15^. Net reclassification improvement (NRI) and integrated discriminatory improvement (IDI) were performed according to the methods described by Pencina *et al*.^16^. Additional analyses were carried out by adding one point for TTR <65% to the ATRIA, ORBIT and HEMORR_2_HAGES scores (as ‘labile INR’ was already included within the HAS-BLED score), in order to determine if this results into an improvement of the predictive ability for major bleeding.

Goodness of fit of the new bleeding risk models was evaluated using the Hosmer-Lemeshow test. Finally, clinical usefulness and net benefit of the new predictive models were estimated using decision curve analyses (DCAs)^17,18^. The DCA test identifies patients who will have any major bleeding, based on the predictions of one risk score when is compared with another score. The x-axis shows threshold values for major bleeding risk while the y-axis represents the net benefit for the different threshold values of major bleeding risk. The prediction models that are the farthest away from the slanted dash grey line (i.e., assume all major bleeding) and the horizontal black line (i.e., assume none major bleeding) demonstrates the higher net benefit.

A p value < 0.05 was accepted as statistically significant. Statistical analyses were performed using SPSS v. 19.0 (SPSS, Inc., Chicago, IL, USA), MedCalc v. 16.4.3 (MedCalc Software bvba, Ostend, Belgium) and STATA v. 12.0 (Stata Corp., College Station, TX, USA) for Windows.

## Results

We included 1361 patients (48.7% male; median age 76, IQR 71–81 years), followed-up for a median of 6.5 years (IQR 4.3–7.9). A summary of clinical characteristics at baseline is shown in Table 1. Median CHA_2_DS_2_-VASc was 4 (IQR 3–5) and the median TTR at 6 months after entry was 80% (IQR 66–100), although 24.2% of patients had a TTR <65%.

**Table 1:** Baseline clinical characteristics.

N = 1361	**HAS-BLED**		*p*	**ATRIA**		*p*	**ORBIT**		*p*	**HEMORR**_**2**_ **HAGES**		*p*
	*score*			*score*			*score*			*score*		
	0–2	≥3		<4	≥4		<3	≥3		0–1	≥2	
	*Low risk*	*High Risk*		*Low risk*	*Medium/High risk*		*Low risk*	*Medium/High risk*		*Low risk*	*Medium/High risk*	
	N = 752	N = 609		N = 1059	N = 302		N = 1042	N = 319		N = 343	N = 1018	
Female sex, n (%)	400 (53.2)	298 (48.9)	0.118	532 (50.2)	166 (55)	0.147	529 (50.8)	150 (47)	0.490	153 (44.6)	545 (53.5)	0.004
Age (years), median (IQR)	75 (68–80)	78 (73–82)	<0.001	75 (69–80)	79 (75–83)	<0.001	75 (69–80)	80 (76–83)	<0.001	70 (64–74)	79 (74–82)	<0.001
**Comorbidities, n (%)**												
Hypertension	545 (72.5)	571 (93.8)	<0.001	850 (80.3)	266 (88.1)	0.002	845 (81.1)	271 (85)	0.116	208 (60.6)	908 (89.2)	<0.001
Diabetes mellitus	171 (22.7)	192 (31.5)	<0.001	267 (25.2)	96 (31.8)	0.023	254 (24.4)	109 (34.2)	0.001	68 (19.8)	295 (29.0)	0.001
Heart failure	212 (28.2)	217 (35.6)	0.003	303 (28.6)	126 (41.7)	<0.001	295 (28.3)	134 (42.0)	<0.001	72 (21)	357 (35.1)	<0.001
History of stroke/TIA	32 (4.3)	225 (36.9)	<0.001	185 (17.5)	72 (23.8)	0.013	174 (16.7)	83 (26.0)	<0.001	10 (2.9)	247 (24.3)	<0.001
Hepatic impairment	2 (0.3)	16 (2.6)	<0.001	14 (1.3)	4 (1.3)	0.997	12 (1.2)	6 (1.9)	0.318	1 (0.3)	17 (1.7)	0.053
Renal impairment	10 (1.3)	134 (22.0)	<0.001	83 (7.8)	61 (20.2)	<0.001	76 (7.3)	68 (21.3)	<0.001	10 (2.9)	134 (13.2)	<0.001
Anaemia	117 (15.6)	137 (22.5)	0.001	13 (1.2)	241 (79.8)	<0.001	36 (3.5)	218 (68.3)	<0.001	4 (1.2)	250 (24.6)	<0.001
Coronary artery disease	95 (12.6)	160 (26.3)	<0.001	181 (17.1)	74 (24.5)	0.004	157 (15.1)	98 (30.7)	<0.001	47 (13.7)	208 (20.4)	0.006
Current smoking habit	101 (13.4)	109 (17.9)	0.023	162 (15.3)	48 (15.9)	0.800	152 (14.6)	58 (18.2)	0.120	52 (15.2)	158 (15.5)	0.873
Current alcohol consumption	4 (0.5)	46 (7.6)	<0.001	41 (3.9)	9 (3.0)	0.468	39 (3.7)	11 (3.4)	0.807	1 (0.3)	49 (4.8)	<0.001
Concomitant antiplatelet treatment	27 (3.6)	216 (35.5)	<0.001	178 (16.8)	65 (21.5)	0.059	139 (13.3)	104 (32.6)	<0.001	9 (2.6)	234 (23.0)	<0.001
Prior malignant disease	47 (6.3)	58 (9.5)	0.024	72 (6.8)	33 (10.9)	0.018	71 (6.8)	34 (10.7)	0.024	5 (1.5)	100 (9.8)	<0.001
Time in therapeutic range (%), median (IQR)	80 (66–100)	64 (50–83)	<0.001	80 (66–100)	80 (60–94)	0.028	80 (66–100)	80 (60–88)	0.003	80 (66–100)	80 (66–100)	0.278
TTR <65%, n (%)	138 (18.4)	311 (51.1)	<0.001	250 (23.6)	80 (26.5)	0.302	237 (22.7)	93 (29.2)	0.019	78 (22.7)	252 (24.8)	0.452
**Thromboembolic risk**												
CHA_2_DS_2_-VASc score, median (IQR)	3 (2–4)	5 (4–6)	<0.001	4 (3–5)	5 (4–6)	<0.001	4 (3–5)	5 (4–6)	<0.001	3 (2–3)	4 (4–5)	<0.001

CHA_2_DS_2_-VASc = cardiac failure or dysfunction, hypertension, age ≥75 [doubled], diabetes, stroke [doubled] – vascular disease, age 65–74 years and sex category [female]; IQR = interquartile range; TIA = transient ischemic attack; TTR = time in therapeutic range.

Median (IQR) values in our cohort for HAS-BLED, ATRIA, ORBIT and HEMORR_2_HAGES were 2 (IQR 2–3), 3 (IQR 1–3), 1 (IQR 1–2) and 2 (IQR 1–3), respectively. Based on HAS-BLED, 44.7% of patients were categorised as being at ‘high risk’ for bleeding, whilst for HEMORR_2_HAGES, ATRIA and ORBIT, the corresponding proportions categorised as ‘medium/high risk’ (i.e. the ‘action needed’ threshold) were 74.8%, 22.3% and 23.4%, respectively.

During the 6.5 years (IQR 4.3–7.9) follow-up, there were 250 (18.4%) major bleeding events (i.e. 2.82%/year), of which 78 (5.7%, i.e. 0.88%/year) were intracranial bleeds and 97 (7.1%, i.e. 1.09%/year) were gastrointestinal bleeds. Fatal bleeds occurred in 52 (3.8%, i.e. 0.59%/year).

### Relationship to comorbidities

Diabetes mellitus, heart failure, coronary artery disease and prior malignancy were generally more prevalent in high risk groups in all scores (Table 1). Patients at medium/high risk of bleeding using HEMORR_2_HAGES were more frequently female (p = 0.004), but there was no sex association with other scores. Anaemia was more prevalent with high risk HAS-BLED (p = 0.001). Patients at medium/high risk according to ORBIT and ATRIA more commonly had previous stroke/TIA. As expected, thromboembolic risk according to CHA_2_DS_2_-VASc score was higher amongst high or medium/high bleeding risk categories (p < 0.001 for all scores).

Median TTR analyzed was significantly lower in the HAS-BLED high risk group (p < 0.001) and ATRIA (p = 0.028) and ORBIT (p = 0.003) medium/high risk groups. The proportion with poor anticoagulation control (TTR <65%) was significantly increased in the high risk HAS-BLED (p < 0.001) and medium/high ORBIT (p = 0.019) categories.

### Bleeding events

Of 250 major bleeding, 65.2% occurred in the HAS-BLED high risk category and 82.4% in the HEMORR_2_HAGES medium/high risk category; in contrast, most major bleeds occurred in ‘low risk’ ATRIA and ORBIT scores, with only 29.6% and 34.0% of major bleeds occurred in their respective ‘medium/high risk’ categories. Odds ratios (OR) for major bleeds using the four bleeding risk scores were calculated. The HAS-BLED high risk category [OR 2.00 (1.51–2.63); p < 0.001] showed the highest value compared with the medium/high risk categories for ATRIA [OR 1.63 (1.20–2.22); p = 0.002], ORBIT [OR 1.93 (1.43–2.60); p < 0.001] and HEMORR_2_HAGES [OR 1.72 (1.21–2.45); p = 0.002] (Table 2).

**Table 2:** Distribution of major bleeding events according to the bleeding risk scores.

Score	Major Bleeding (N = 250)			
	HAS-BLED N (%)	ATRIA N (%)	ORBIT N (%)	HEMORR2HAGES N (%)
0	5 (2.0)	14 (5.6)	43 (17.2)	7 (2.8)
1	20 (8.0)	56 (22.4)	71 (28.4)	37 (14.8)
2	62 (24.8)	21 (8.4)	51 (20.4)	55 (22.0)
3	82 (32.8)	85 (34.0)	38 (15.2)	64 (25.6)
4	49 (19.6)	27 (10.8)	27 (10.8)	44 (17.6)
≥5	30 (12.8)	47 (18.8)	20 (8.0)	43 (17.2)
Low Risk	87 (34.8)	176 (70.4)	165 (66.0)	44 (17.6)
High or Medium/High Risk	163 (65.2)	74 (29.6)	85 (34.0)	206 (82.4)
OR (95% CI), p*	2.00 (1.51–2.63), p < 0.001	1.63 (1.20–2.22), p = 0.002	1.93 (1.43–2.60), p < 0.001	1.72 (1.21–2.45), p = 0.002

CI = confidence interval; OR = odds ratio. *For the high or medium/high risk category.

**Figure 1:**
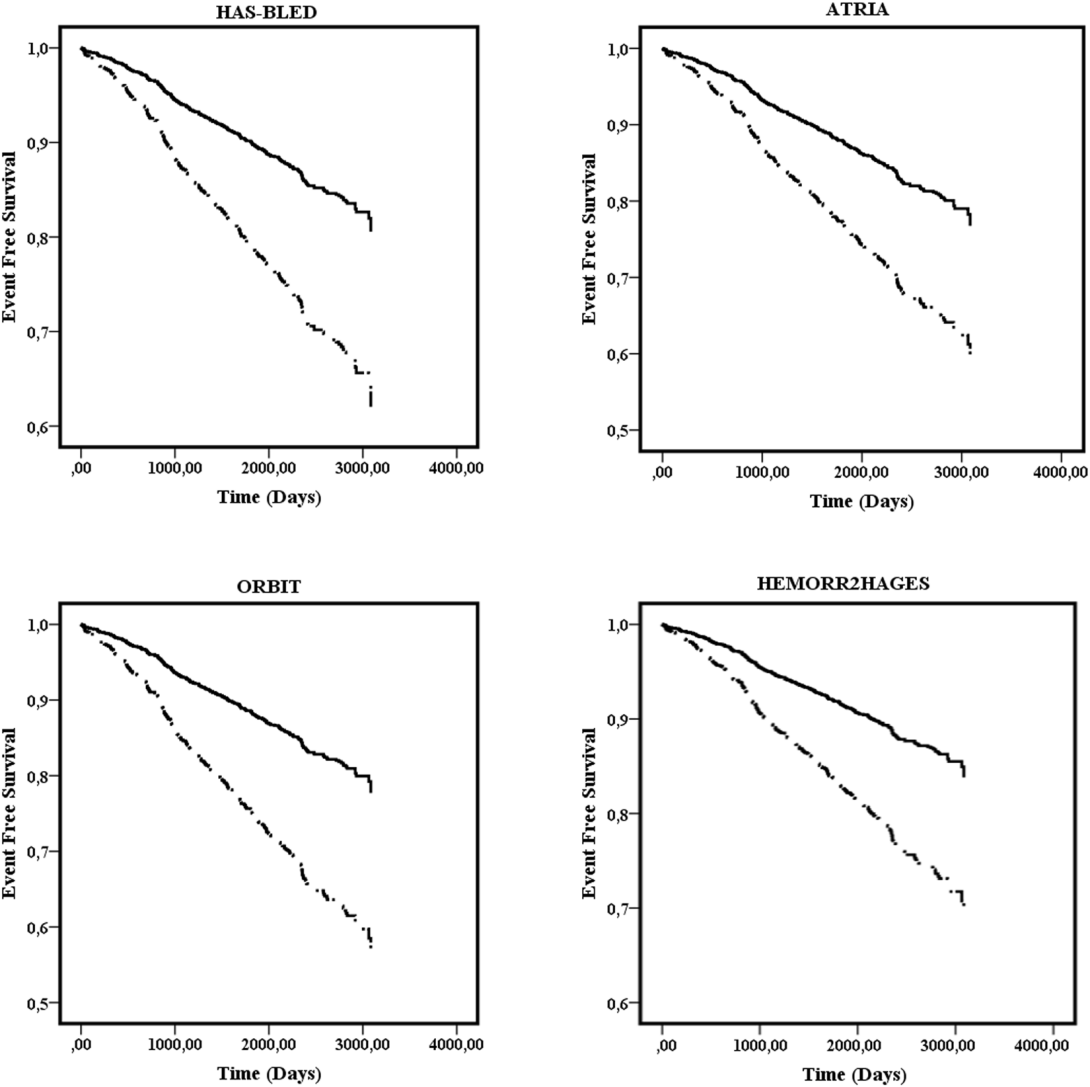
Event free survival for major bleeding according to risk categories for each bleeding risk score.

Univariate Cox regression analysis also showed a significant association between the four bleeding risk scores and major bleeds, whether analysed as continuous or categorical variables (Table 3). Survival analysis demonstrated that patients categorized at high risk or medium/high risk showed an increased risk of major bleeding (HAS-BLED: Log-Rank 40.24, p < 0.001; ATRIA: Log-Rank 25.82, p < 0.001; ORBIT: Log-Rank 40.88, p < 0.001 and HEMORR_2_HAGES: Log-Rank 21.33, p < 0.001) (Fig. 1).

**Table 3:** Univariate Cox regression analysis between bleeding risk scores and major bleeding events.

	HR	95% CI	*p*
HAS-BLED (*as continuous*)	1.66*	1.50–1.83	<0.001
HAS-BLED (*as categorical*)	3.00	2.31–3.89	<0.001
ATRIA (*as continuous*)	1.20*	1.13–1.28	<0.001
ATRIA (*as categorical*)	2.00	1.52–2.63	<0.001
ORBIT (*as continuous*)	1.37*	1.27–1.49	<0.001
ORBIT (*as categorical*)	2.30	1.77–2.99	<0.001
HEMORR_2_HAGES (*as continuous*)	1.43*	1.32–1.56	<0.001
HEMORR_2_HAGES (*as categorical*)	2.12	1.53–2.94	<0.001

CI = confidence interval; HR = hazard ratio. *As per every score point.

Receiver operating characteristic (ROC) curves analysis shows that all scores predicted major bleeding in patients with AF, with c-indexes of 0.62 (p < 0.001) for HAS-BLED, and 0.54 (p = 0.004), 0.56 (p < 0.001) and 0.54 (p = 0.007) for ATRIA, ORBIT and HEMORR_2_HAGES (Supplementary Table 2), with HAS-BLED having the best predictive value. Comparison of the ROC curves according to DeLong *et al*.^15^ demonstrated that HAS-BLED had the best performance of the four scores (Table 4).

**Table 4:** Comparison of the ROC curves, IDI and NRI of the four bleeding risk scores.

HAS-BLED *vs*.	z statistic*	*p*	IDI	*p*	NRI	*p*
ATRIA	4.017	<0.001	0.0309	0.142	0.1598	<0.001
ORBIT	2.510	<0.001	0.0240	0.067	0.1212	0.007
HEMORR_2_HAGES	2.136	<0.001	0.0311	0.347	0.1574	<0.001

IDI = integrated discriminatory improvement; NRI = net reclassification improvement. *For c-index comparison.

**Figure 2:**
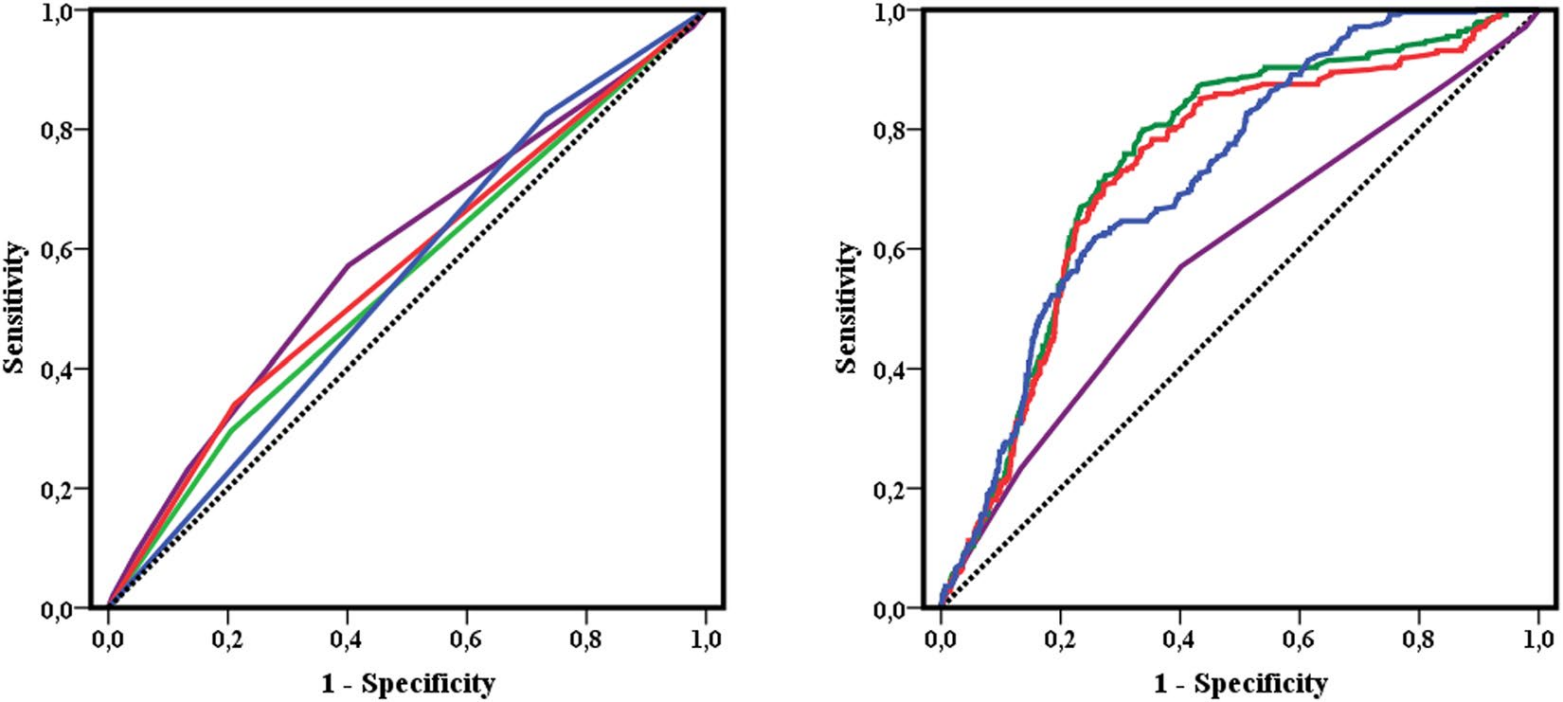
ROC curves for original and modified bleeding risk scores (adding TTR <65%).

When labile INR or poor anticoagulation control (i.e. TTR <65%) was added to ATRIA, ORBIT and HEMORR_2_HAGES, this modification significantly increased the ability of discrimination and their predictive values (Table 5). Comparison of the original and modified scores demonstrated significant improvements in c-indexes for the ATRIA, ORBIT and HEMORR_2_HAGES modified scores (p < 0.001 for the three scores). Reclassification analysis showed an improvement in sensitivity and significant positive reclassification of the modified scores compared with the original, based on the IDI and NRI (Table 5; Fig. 2). Based on the p values of the Hosmer-Lemeshow test, the new predictive models that include poor anticoagulation control (TTR <65%) were properly calibrated (ATRIA, p = 0.981; ORBIT, p = 0.569 and HEMORR_2_HAGES, p = 0.294).

**Table 5:** Comparison of the ROC curves, IDI and NRI of the modified bleeding risk scores (by addition of labile INR defined as time in therapeutic range <65%).

	**C-index**	**95% CI**	z statistic*	***p***	**IDI**	***p***	**NRI**	***p***
*vs*. ATRIA								
ATRIA + TTR <65%	0.751	0.727–0.774	7.514	<0.001	0.0326	<0.001	0.1527	<0.001
*vs*. ORBIT								
ORBIT + TTR <65%	0.733	0.709–0.757	5.087	<0.001	0.0270	<0.001	0.1097	<0.001
*vs*. HEMORR_2_HAGES								
HEMORR_2_HAGES + TTR <65%	0.729	0.704–0.752	4.689	<0.001	0.0159	<0.001	0.0598	0.007

CI = confidence interval; IDI = integrated discriminatory improvement; NRI = net reclassification index; TTR = time in therapeutic range. *For c-index comparison.

**Figure 3:**
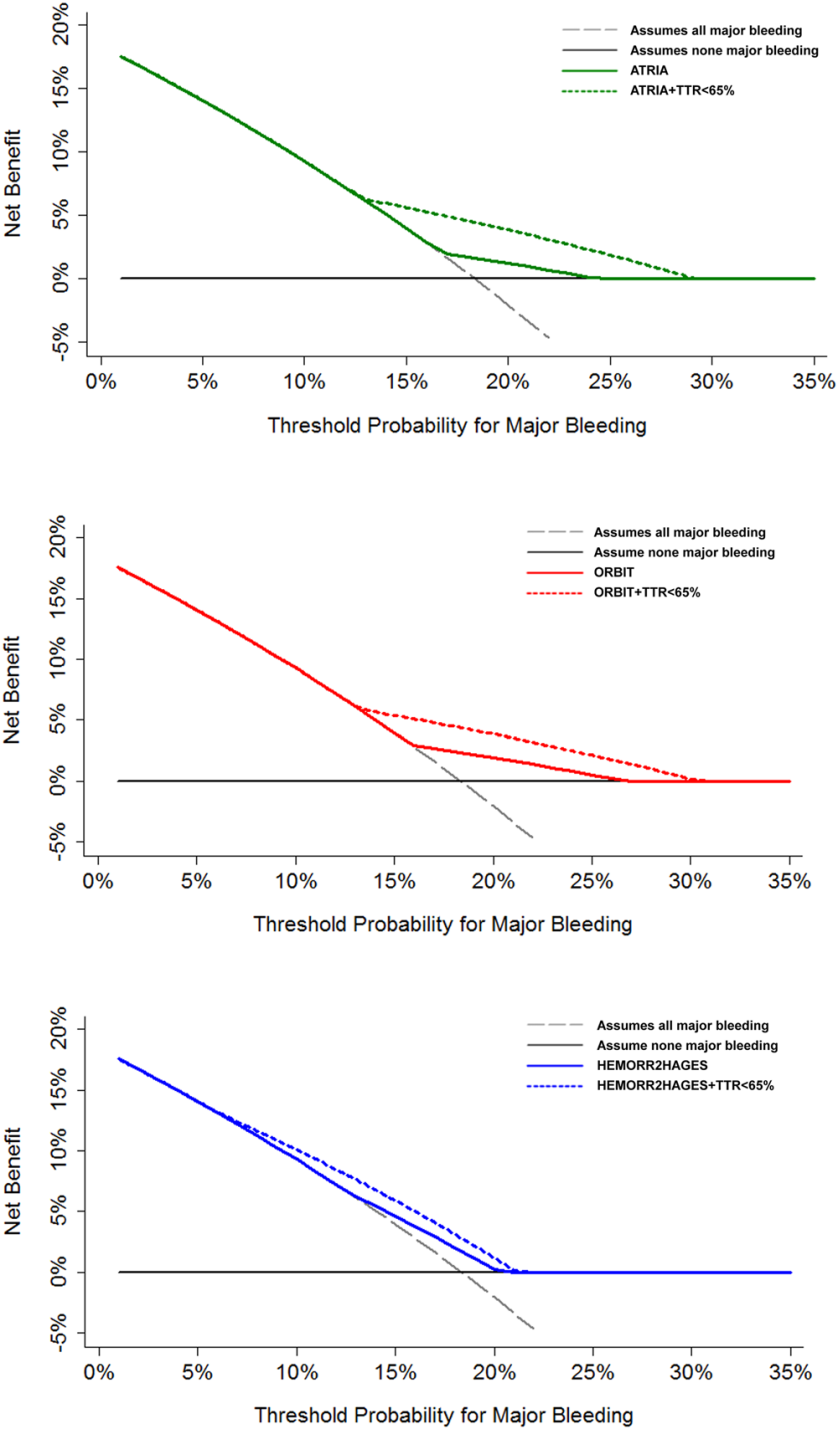
Decision curves for the original and modified bleeding risk scores (adding TTR <65%).

Finally, decision curve analysis (DCA) graphically demonstrates that the overall risk of major bleeding is 19%, based on the intersection of the y-axis and the slanted dash grey line. As they are farthest away from the slanted dash grey line (i.e., assume all major bleeding) and the horizontal black line (i.e., assume none major bleeding), the modified ATRIA, ORBIT and HEMORR_2_HAGES scores (that include labile INR) demonstrates improved clinical usefulness and a higher net benefit compared to the original scores (Fig. 3).

## Discussion

In this ‘real world’ study, our principal finding was that in AF patients taking VKAs, HAS-BLED, ATRIA, ORBIT and HEMORR_2_HAGES scores are all associated with major bleeding, although HAS-BLED had the best predictive ability. Second, adding labile INR (TTR <65%) to ATRIA, ORBIT and HEMORR_2_HAGES scores significantly improved their predictive value for major bleeding, suggesting that these three scores would perform suboptimally in VKA users by not considering ‘labile INR’ as a criterion for bleeding. Indeed, the modified ATRIA, ORBIT and HEMORR_2_HAGES scores (that include labile INR) demonstrated improved clinical usefulness and a higher net benefit compared to the original scores.

Given that the VKAs are the commonest OACs in use world-wide, our findings have major implications for bleeding risk assessment in relation to OAC use. Also bleeding risk is not a ‘static’ process, and patients require re-evaluation at every opportunity over the course of the patient pathway^19^. The appropriate use of bleeding risk scores has been discussed, and these scores are to ‘flag up’ patients potentially at risk of bleeding for more careful review and follow-up. Thus, the ATRIA and ORBIT categorise most patients at ‘low risk’ and hence, would not have ‘flag up’ patients potentially at risk of bleeding – indeed, most patients sustaining major bleeding events occurred in the ‘low risk’ categories of the ATRIA and ORBIT scores.

Given that bleeding risk can be modified, appropriate use of bleeding scores should be to focus attention on reversible bleeding risk factors, such as uncontrolled hypertension, excess alcohol and concomitant antiplatelet therapy or NSAID use, as well as labile INRs in a patient taking VKA^19^. These features are fulfilled by HAS-BLED which has been validated in patients on anticoagulants (whether VKA or non-VKA), aspirin or no antithrombotic therapy – hence, the validity of using this bleeding score in all steps of the patient management pathway.

In the present study, all four bleeding risk scores were associated with major bleeding, although HAS-BLED had the best predictive performance, based on the c-index and NRI. Indeed, HAS-BLED has previously demonstrated better prediction than ATRIA and HEMORR_2_HAGES, even for intracranial haemorrhage^5,20–26^.

The ATRIA (Anticoagulation and Risk Factors in Atrial Fibrillation) score was proposed in 2011 to predict bleeding associated with warfarin^27^, but none of the risk score criteria includes assessment of quality of anticoagulation control or the concomitant use of antiplatelet therapy. As previously described, the HAS-BLED score has a better performance than ATRIA amongst anticoagulated patients with AF, whether with VKA^23,25^ or non-VKA anticoagulants^28^, as well as amongst non-anticoagulated patients^29^. More recently, the ORBIT score was derived from an industry-sponsored registry and proposed as a simple score to assess the risk of bleeding in patients with AF regardless of the type of anticoagulant, whether VKA or non-VKA^30^. Although the ORBIT score predicted major bleeding in the large cohort of the ROCKET-AF trial^31,32^, the ORBIT score ignores the quality of anticoagulation control as a criterion and has been shown to be inferior to HAS-BLED in predicting bleeding amongst AF patients on VKA and non-VKA anticoagulants^33–35^, as well as those who are non-anticoagulated^29^.

The association between the TTR and adverse events has been shown in numerous studies. For example, an increased risk of major bleeds has been consistently shown in patients with VKAs with a TTR below than 65%^36–39^. Many other clinical factors have been added into bleeding risk stratification schemes, but these have been based on complex scoring systems derived from multivariate analyses and thus, difficult to apply in clinical daily practice^6^. Whilst undoubtedly interesting and necessary to develop risk scores for assessing the risk of bleeding irrespective of the anticoagulant and make these scores as simple as possible, the VKAs are still the most commonly used OAC worldwide, and thus, anticoagulation control is an issue that cannot be ignored to support the appropriate clinical decision making. Given the close relationship of bleeding to labile INRs and poor TTR, attention to this clinical factor amongst those patients taking a VKA is crucial^7^. Of note, ‘labile INR’ can also be easily defined using other simple (and easily accessible) parameters, such as the proportion of INRs in range, INR variability, time above range, INR >5 twice, INR >8 once, or INR <2 twice, etc.^13,40^. The results of the present study reinforce this perspective, since the inclusion of ‘labile INR’ into the ATRIA, ORBIT and HEMORR_2_HAGES would significantly increase the predictive ability and clinical usefulness of these scores. Importantly, this suggests that these scores perform suboptimally in VKA patients unless labile INR is considered. Hence, these findings observed in ‘real world’ AF patients support the results from clinical trial cohorts^33,35^.

### Limitations

This study is limited by its single centre design, with a Caucasian based population. The dataset was collected prospectively, although we calculated some risk scores (CHA_2_DS_2_-VASc, HAS-BLED, ATRIA and ORBIT) and performed the analyses retrospectively, since at the time of patient inclusion these newer scores were not yet described and hence, were not used to ‘clinically manage’ these patients. At the beginning of the study all patients were treated with acenocoumarol, which has a shorter half-life than other VKAs. However, one strength of our study is the inclusion of consecutive AF patients that were stable with VKA (INR 2.0–3.0) for at least 6 months. Follow-up was also done in an anticoagulation clinic, where at the beginning of OAC therapy patients are carefully followed, according to a standardized care protocol. This aspect may have minimized our bleeding events, and the generalizability to other settings with less intense follow-up.

## Conclusions

In AF patients taking VKAs, the HAS-BLED score had the best predictive ability. Adding labile INR (TTR <65%) to ATRIA, ORBIT and HEMORR_2_HAGES scores improved their predictive value for major bleeding leading to improved clinical usefulness and a higher net benefit compared to the original scores. This suggests that these three scores would perform suboptimally in VKA users by not considering ‘labile INR’ as a criterion for bleeding.

## Electronic supplementary material

Supplementary Material

## References

[1] Hart RG, Pearce LA, Aguilar MI (2007). Meta-analysis: antithrombotic therapy to prevent stroke in patients who have nonvalvular atrial fibrillation. Ann Intern Med.

[2] Medi C, Hankey GJ, Freedman SB (2010). Stroke risk and antithrombotic strategies in atrial fibrillation. Stroke.

[3] Task-Force-Members. *et al*. Focused update of the ESC Guidelines for the management of atrial fibrillation: an update of the 2010 ESC Guidelines for the management of atrial fibrillation *Developed with the special contribution of the European Heart Rhythm Association. *Eur Heart J* **33**, 2719–2747 (2012). 10.1093/eurheartj/ehs253 22922413

[4] Wan Y (2008). Anticoagulation control and prediction of adverse events in patients with atrial fibrillation: a systematic review. Circ Cardiovasc Qual Outcomes.

[5] Zhu W, He W, Guo L, Wang X, Hong K (2015). The HAS-BLED Score for Predicting Major Bleeding Risk in Anticoagulated Patients With Atrial Fibrillation: A Systematic Review and Meta-analysis. Clin Cardiol.

[6] Gallego P (2012). Relation of the HAS-BLED bleeding risk score to major bleeding, cardiovascular events, and mortality in anticoagulated patients with atrial fibrillation. Circ Arrhythm Electrophysiol.

[7] Lip GY, Lane DA (2015). Assessing bleeding risk in atrial fibrillation with the HAS-BLED and ORBIT scores: clinical application requires focus on the reversible bleeding risk factors. Eur Heart J.

[8] Lip GY (2015). Assessing Bleeding Risk With the HAS-BLED Score: Balancing Simplicity, Practicality, and Predictive Value in Bleeding-Risk Assessment. Clin Cardiol.

[9] De Caterina R (2013). Vitamin K antagonists in heart disease: current status and perspectives (Section III). Position paper of the ESC Working Group on Thrombosis–Task Force on Anticoagulants in Heart Disease. Thromb Haemost.

[10] White HD (2007). Comparison of outcomes among patients randomized to warfarin therapy according to anticoagulant control: results from SPORTIF III and V. Arch Intern Med.

[11] Apostolakis S, Sullivan RM, Olshansky B, Lip GY (2013). Factors affecting quality of anticoagulation control among patients with atrial fibrillation on warfarin: the SAMe-TT(2)R(2) score. Chest.

[12] Rosendaal FR, Cannegieter SC, van der Meer FJ, Briet E (1993). A method to determine the optimal intensity of oral anticoagulant therapy. Thromb Haemost.

[13] National-Institute-for-Health-and-Care-Excellence. Atrial fibrillation: the management of atrial fibrillation. London: National Clinical Guideline Centre (2014).

[14] Schulman S, Kearon C (2005). Definition of major bleeding in clinical investigations of antihemostatic medicinal products in non-surgical patients. J Thromb Haemost.

[15] DeLong ER, DeLong DM, Clarke-Pearson DL (1988). Comparing the areas under two or more correlated receiver operating characteristic curves: a nonparametric approach. Biometrics.

[16] Pencina, M. J., D’Agostino, R. B. Sr., D’Agostino, R. B. Jr. & Vasan, R. S. Evaluating the added predictive ability of a new marker: from area under the ROC curve to reclassification and beyond. *Stat Med* **27**, 157–172; discussion 207–112 (2008). 10.1002/sim.2929 17569110

[17] Vickers AJ, Cronin AM, Elkin EB, Gonen M (2008). Extensions to decision curve analysis, a novel method for evaluating diagnostic tests, prediction models and molecular markers. BMC Med Inform Decis Mak.

[18] Vickers AJ, Elkin EB (2006). Decision curve analysis: a novel method for evaluating prediction models. Med Decis Making.

[19] Lip GY, Lane DA (2016). Bleeding risk assessment in atrial fibrillation: observations on the use and misuse of bleeding risk scores. J Thromb Haemost.

[20] Thomas IC, Sorrentino MJ (2014). Bleeding risk prediction models in atrial fibrillation. Curr Cardiol Rep.

[21] Abumuaileq RR (2014). Comparative evaluation of HAS-BLED and ATRIA scores by investigating the full potential of their bleeding prediction schemes in non-valvular atrial fibrillation patients on vitamin-K antagonists. Int J Cardiol.

[22] Caldeira D, Costa J, Fernandes RM, Pinto FJ, Ferreira JJ (2014). Performance of the HAS-BLED high bleeding-risk category, compared to ATRIA and HEMORR2HAGES in patients with atrial fibrillation: a systematic review and meta-analysis. J Interv Card Electrophysiol.

[23] Apostolakis S, Lane DA, Guo Y, Buller H, Lip GY (2012). Performance of the HEMORR(2)HAGES, ATRIA, and HAS-BLED bleeding risk-prediction scores in patients with atrial fibrillation undergoing anticoagulation: the AMADEUS (evaluating the use of SR34006 compared to warfarin or acenocoumarol in patients with atrial fibrillation) study. J Am Coll Cardiol.

[24] Fauchier L (2016). Predictive ability of HAS-BLED, HEMORR2HAGES, and ATRIA bleeding risk scores in patients with atrial fibrillation. A French nationwide cross-sectional study. Int J Cardiol.

[25] Roldan V (2013). Predictive value of the HAS-BLED and ATRIA bleeding scores for the risk of serious bleeding in a “real-world” population with atrial fibrillation receiving anticoagulant therapy. Chest.

[26] Lip GY (2012). Assessing the risk of bleeding in patients with atrial fibrillation: the Loire Valley Atrial Fibrillation project. Circ Arrhythm Electrophysiol.

[27] Fang MC (2011). A new risk scheme to predict warfarin-associated hemorrhage: The ATRIA (Anticoagulation and Risk Factors in Atrial Fibrillation) Study. J Am Coll Cardiol.

[28] Apostolakis S, Lane DA, Guo Y, Buller H, Lip GY (2013). Performance of the HEMORR 2 HAGES, ATRIA, and HAS-BLED bleeding risk-prediction scores in nonwarfarin anticoagulated atrial fibrillation patients. J Am Coll Cardiol.

[29] Guo Y-t (2016). Assessing bleeding risk in 4824 Asian patients with atrial fibrillation: The Beijing PLA Hospital Atrial Fibrillation Project. Sci Rep.

[30] O’Brien EC (2015). The ORBIT bleeding score: a simple bedside score to assess bleeding risk in atrial fibrillation. Eur Heart J.

[31] Lip GY, Lane DA (2016). ACP Journal Club. The 5-factor ORBIT bleeding score predicted major bleeding at 2 years in patients with atrial fibrillation. Ann Intern Med.

[32] Patel MR (2011). Rivaroxaban versus warfarin in nonvalvular atrial fibrillation. N Engl J Med.

[33] Proietti M, Senoo K, Lane DA, Lip GY (2016). Major Bleeding in Patients with Non-Valvular Atrial Fibrillation: Impact of Time in Therapeutic Range on Contemporary Bleeding Risk Scores. Sci Rep.

[34] Senoo K, Lip GYH (2016). Predictive abilities of the HAS-BLED and ORBIT bleeding risk scores in non-warfarin anticoagulated atrial fibrillation patients: An ancillary analysis from the AMADEUS trial. Intern J Cardiol.

[35] Senoo K, Proietti M, Lane DA, Lip GY (2016). Evaluation of the HAS-BLED, ATRIA, and ORBIT Bleeding Risk Scores in Patients with Atrial Fibrillation Taking Warfarin. Am J Med.

[36] Gallego P (2013). Cessation of oral anticoagulation in relation to mortality and the risk of thrombotic events in patients with atrial fibrillation. Thromb Haemost.

[37] Roldán V, Marín F (2015). The importance of excellence in the quality of anticoagulation control whilst taking vitamin K antagonists. Thromb Haemost.

[38] Gallagher A, Setakis E, Plumb J, Clemens A, van Staa T (2011). Risk of stroke and mortality associated with suboptimal anticoagulation in atrial fibrillation patients. Thromb Haemost.

[39] Pokorney SD (2015). Patients’ time in therapeutic range on warfarin among US patients with atrial fibrillation: Results from ORBIT-AF registry. Am Heart J.

[40] Ruiz-Ortiz M, Bertomeu V, Cequier A, Marin F, Anguita M (2015). Validation of the SAMe-TT2R2 score in a nationwide population of nonvalvular atrial fibrillation patients on vitamin K antagonists. Thromb Haemost.

