# Supplementary material for: Importance of time in therapeutic range on bleeding risk prediction using clinical risk scores in patients with atrial fibrillation
Source: Sci Rep. 2017 Sep 21;7:12066. doi: 10.1038/s41598-017-11683-2 (PMC5608893; doi:10.1038/s41598-017-11683-2)
Supplement: Supplementary file 1 — Supplementary Material [file 41598_2017_11683_MOESM1_ESM.doc]

**Importance of time in therapeutic range on bleeding risk prediction using clinical risk scores in patients with atrial fibrillation**

José Miguel Rivera-Caravaca^1^, Vanessa Roldán^2*^, María Asunción Esteve-Pastor^1^, Mariano Valdés^1^, Vicente Vicente^2^, Gregory YH Lip^3†^, Francisco Marín^1†^

[^†^joint senior authors]

1. Department of Cardiology, Hospital Clínico Universitario Virgen de la Arrixaca, Instituto Murciano de Investigación Biosanitaria (IMIB-Arrixaca), Murcia, Spain.
2. Department of Hematology and Clinical Oncology, Hospital Universitario Morales Meseguer, University of Murcia, Instituto Murciano de Investigación Biosanitaria (IMIB-Arrixaca), Murcia, Spain.
3. University of Birmingham Institute of Cardiovascular Sciences. City Hospital, University of Birmingham, Birmingham, United Kingdom, and Aalborg Thrombosis Research Unit, Department of Clinical Medicine, Aalborg University, Aalborg, Denmark.

**Address for correspondence**

Vanessa Roldán, MD, PhD

Department of Hematology and Clinical Oncology

Hospital Universitario Morales Meseguer

Avda. Marqués de los Vélez s/n 30008, Murcia, Spain

Tel./fax: +34 968 36 09 00; E-mail: [vroldans@um.es](mailto:vroldans@um.es)

**Supplementary Material**

**Supplementary Table 1. Clinical criteria of the various bleeding risk scores**

| **Bleeding Risk Scores** | **Criteria** | **Score** |
| --- | --- | --- |
| HAS-BLED | Uncontrolled hypertension (systolic blood pressure >160 mmHg) | 1 |
|  | Impaired renal function (dialysis, transplant, Cr >2.26 mg/dL or >200 µmol/L) or impaired liver function (cirrhosis or bilirubin >2x normal or AST/ALT/AP >3x normal) | 1 or 2 |
|  | History of stroke | 1 |
|  | History of bleeding | 1 |
|  | Labile international normalized ratio (INR) (< 60% of time in therapeutic range) | 1 |
|  | Age >65 years | 1 |
|  | Concomitant use of antiplatelet agents or non-steroidal anti-inflammatory drugs and alcohol consumption (more than 8 units/week) | 1 or 2 |

| ATRIA | Anemia | 3 |
| --- | --- | --- |
|  | Severe renal impairment (GFR <30 mL/min or dialysis-dependent) | 3 |
|  | Age ≥75 years | 2 |
|  | Any prior hemorrhage diagnosis | 1 |
|  | Hypertension history | 1 |
|  | | |
| ORBIT | Age ≥75 years | 1 |
|  | Reduced haemoglobin/anemia | 2 |
|  | History of bleeding | 2 |
|  | Impaired renal function (<60 mL/min/1.73 m^2^) | 1 |
|  | Treatment with antiplatelets | 1 |

| HEMORR_2_HAGES | Hepatic or renal disease | 1 |
| --- | --- | --- |
|  | Alcohol abuse | 1 |
|  | Malignancy history | 1 |
|  | Age >75 years | 1 |
|  | Reduced platelet count or function (includes aspirin use, any thrombocytopenia or blood dyscrasia, like hemophilia) | 1 |
|  | History of bleeding | 2 |
|  | Uncontrolled hypertension | 1 |
|  | Anemia | 1 |
|  | Genetic factors (CYP 2C9 single-nucleotide polymorphisms) | 1 |
|  | Excessive Fall Risk | 1 |
|  | History of stroke | 1 |

**Supplementary Table 2. Predictive values for the four bleeding risk scores.**

|  | **C-index** | **95% CI** | **z statistic** | ***p*** |
| --- | --- | --- | --- | --- |
| HAS-BLED | 0.625 | 0.599-0.651 | 7.461 | <0.001 |
| ATRIA | 0.545 | 0.518-0.572 | 2.894 | 0.004 |
| ORBIT | 0.565 | 0.538-0.591 | 3.991 | <0.001 |
| HEMORR_2_HAGES | 0.547 | 0.520-0.573 | 3.379 | 0.007 |
| CI = confidence interval. | | | | |
